# Supplementary material for: syntenet: an R/Bioconductor package for the inference and analysis of synteny networks
Source: Bioinformatics. 2022 Dec 20;39(1):btac806. doi: 10.1093/bioinformatics/btac806 (PMC9825758; doi:10.1093/bioinformatics/btac806)
Supplement: btac806_Supplementary_Data [file btac806_supplementary_data.pdf]

Supplementary Material - syntenet: an R/Bioconductor package for the inference and analysis of synteny networks

Fabricio Almeida-Silva [1,2]**, *Tao Zhao*** [3]**, *Kristian K Ullrich*** [4]**, *M. Eric Schranz*** [5]**, and *Yves Van de Peer*** [1,2,6,7]

[1]VIB-UGent Center for Plant Systems Biology, Ghent, Belgium
[2]Department of Plant Biotechnology and Bioinformatics, Ghent University, Ghent, Belgium
[3]State Key Laboratory of Crop Stress Biology for Arid Areas/Shaanxi Key Laboratory of Apple, College of Horticulture, Northwest AF University, Yangling, China
[4]Department of Evolutionary Biology, Max Planck Institute For Evolutionary Biology, Ploen, Germany
[5]Biosystematics Group, Wageningen University and Research, Wageningen, The Netherlands
[6]College of Horticulture, Academy for Advanced Interdisciplinary Studies, Nanjing Agricultural University, Nanjing, China
[7]Center for Microbial Ecology and Genomics, Department of Biochemistry, Genetics and Microbiology, University of Pretoria, Pretoria, South Africa

18 November 2022

Contents

Introduction 2

Section 1: Recreating phylogenomic profiles from Zhao and Schranz (2019) 2

Section 2: Rebuilding the angiosperm phylogeny from Zhao et al. (2021) 8

Section 3: Exploring synteny networks for Chlorophyta genomes 14

Benchmarking runtime for increasingly large data sets 18

Session information 21

References 24

Introduction

Here, we will use *syntenet* to reproduce the findings from two of our previous papers on synteny networks:

1. Zhao, T., & Schranz, M. E. (2019). Network-based microsynteny analysis identifies major differences and genomic outliers in mammalian and angiosperm genomes. **Proceedings of the National Academy of Sciences**, 116(6), 2165-2174. DOI: 10.1073/pnas.1801757116
2. Zhao, T., Zwaenepoel, A., Xue, J. Y., Kao, S. M., Li, Z., Schranz, M. E., & Van de Peer, Y. (2021). Whole-genome microsynteny-based phylogeny of angiosperms. **Nature Communications**, 12(1), 1-14. DOI: 10.1038/s41467-021-23665-0

Besides, we will run the whole pipeline on a new data set of algae genomes from the Chlorophyta clade. Data were obtained from Pico-PLAZA 3.0 (Van Bel et al. 2018).

```
library(syntenet)
library(tidyverse)
set.seed(123)
```

Section 1: Recreating phylogenomic profiles from Zhao and Schranz (2019)

In this section, we will identify phylogenomic profiles from synteny clusters of BUSCO genes in angiosperms. Then, we will represent the phylogenomic profiles as in Figure 1B of the manuscript. The data for this section were obtained from the original publication (Zhao and Schranz 2019), in its Dataverse repository (DOI: 10.7910/DVN/BDMA7A).

We will start by reading the network as an edge list.

```
#----Download file-------------------------------------------------------------
net_file <- file.path(tempdir(), "busco_network.txt.gz")
if(!file.exists(net_file)) {
    download.file(
        file.path(
            "https://dataverse.harvard.edu/api/access/datafile/",
            ":persistentId?persistentId=doi:10.7910/DVN/BDMA7A/7JWAFI"
        ),
        destfile = net_file
    )
}

#----Read file-----------------------------------------------------------------
busco_network <- readr::read_delim(
    net_file, show_col_types = FALSE, col_names = FALSE, delim = " "
)

head(busco_network)
## # A tibble: 6 x 2
##   X1            X2
```

```
##   <chr>          <chr>
## 1 aar_AA1G00152 bvu_Bv7_172870_irmg
## 2 aar_AA1G00152 csi_1g021926
## 3 aar_AA1G00152 csi_1g021868
## 4 aar_AA1G00152 dca_018939
## 5 aar_AA1G00152 pbr_028008.1
## 6 aar_AA1G00152 pbr_042334.1
```

In this network, node names already contain acronyms (abbreviations) representing each species. To make visualizations more meaningful, we will create a data frame of species metadata containing species names, their corresponding abbreviations, and taxonomic information.

```
## Create a data frame of taxonomic information for each species
### Family and abbreviations
species_metadata <- data.frame(
    Species = c(
        "Vigna radiata", "Vigna angularis", "Phaseolus vulgaris",
        "Glycine max", "Cajanus cajan", "Trifolium pratense",
        "Medicago truncatula", "Arachis duranensis", "Lotus japonicus",
        "Lupinus angustifolius", "Cicer arietinum",
        "Prunus mume", "Prunus persica", "Pyrus x bretschneideri",
        "Malus domestica", "Rubus occidentalis", "Fragaria vesca",
        "Morus notabilis", "Ziziphus jujuba", "Humulus lupulus",
        "Jatropha curcas", "Manihot esculenta", "Ricinus communis",
        "Linum usitatissimum", "Populus trichocarpa", "Cucumus sativus",
        "Cucumis melo", "Citrullus lanatus",
        "Castanea mollissima", "Juglans regia", "Betula pendula",
        "Capsella grandiflora", "Capsella rubella", "Arabidopsis lyrata",
        "Arabidopsis thaliana", "Camelina sativa", "Brassica oleraceae",
        "Brassica rapa", "Brassica napus", "Raphanus raphanistrum",
        "Thellungiella halophila", "Thellungiella salsuginea",
        "Leavenworthia alabamica", "Aethionema arabicum",
        "Schrenkiella parvula", "Boechera stricta", "Arabis alpina",
        "Sisymbrium irio", "Cleome gynandra", "Tarenaya hassleriana",
        "Carica papaya", "Gossypium raimondii", "Theobroma cacao",
        "Eucalyptus grandis", "Citrus sinensis", "Vitis vinifera",
        "Solanum pennellii", "Solanum lycopersicum", "Solanum tuberosum",
        "Solanum melongena", "Capsicum annuum", "Nicotiana benthamiana",
        "Nicotiana tomentosiformis", "Nicotiana attenuata",
        "Nicotiana sylvestris", "Petunia axillaris",
        "Ipomoea nil", "Utricularis gibba", "Sesamum indicum",
        "Mimulus guttatus", "Coffea canephora", "Lactuca sativa",
        "Helianthus annuus", "Daucus carota",
        "Actinidia chinensis", "Chenopodium quinoa", "Spinacia oleraceae",
        "Beta vulgaris", "Amaranthus hypochondriacus",
        "Nelumbo nucifera", "Triticum urartu", "Triticum aestivum",
        "Aegilops tauschii", "Hordeum vulgare", "Brachypodium distachyon",
        "Oryza glaberrima", "Oryza sativa", "Oryza rufipogon",
        "Leersia perrieri", "Phylostachys heterocycla", "Zea mays",
        "Zea mays V4", "Sorghum bicolor", "Setaria italica",
```

```
            "Oropetium thomaeum", "Ananas comosus", "Elaeis guineensis",
            "Phoenix dactylifera", "Musa acuminata", "Dendrobium catenatum",
            "Phalaenopsis equestris", "Asparagus officinalis", "Xerophyta viscosa",
            "Spirodela polyrhiza", "Lemna minor", "Zostera marina",
            "Amborella trichopoda"
        ),
        Abbrev = c(
            "vra", "van", "pvu", "gma", "cca", "tpr", "mtr", "adu", "lja", "Lang",
            "car", "pmu", "ppe", "pbr", "mdo", "roc", "fve", "Mnot", "Zjuj",
            "hlu", "jcu", "mes", "rco", "lus", "ptr", "csa", "cme", "cla",
            "cmo", "jre", "Bpen", "cgr", "cru", "Alyr", "ath", "Csat", "bol",
            "bra", "bnp", "rra", "thh", "tsa", "lal", "aar", "spa", "Bostr", "Alp",
            "sir", "cgy", "tha", "cpa", "gra", "tca", "egr", "csi", "vvi",
            "spe", "sly", "stu", "sme", "can", "nbe", "Ntom", "Natt",
            "Nsyl", "pax", "Inil", "ugi", "sin", "mgu", "coc", "Lsat", "hel",
            "dca", "ach", "Cqui", "sol", "bvu", "Ahyp", "nnu", "tur", "tae", "ata",
            "HORVU", "bdi", "ogl", "osa", "oru", "lpe", "phe", "zma", "Zmay",
            "sbi", "sit", "oth", "aco", "egu", "Pdac", "mac", "Dcat", "peq",
            "Aoff", "Xvis", "spo", "lmi", "zom", "atr"
        ),
        Family = c(
            rep("Fabaceae", 11), rep("Rosaceae", 6),
            "Moraceae", "Rhamnaceae", "Cannabaceae",
            rep("Euphorbiaceae", 3), "Linaceae", "Salicaceae",
            rep("Cucurbitaceae", 3), "Fagaceae", "Juglandaceae", "Betulaceae",
            rep("Brassicaceae", 17), rep("Cleomaceae", 2), "Caricaceae",
            rep("Malvaceae", 2), "Myrtaceae", "Rutaceae", "Vitaceae",
            rep("Solanaceae", 10), "Convolvulaceae", "Lentibulariaceae",
            "Pedaliaceae", "Phrymaceae", "Rubiaceae", rep("Asteraceae", 2),
            "Apiaceae", "Actinidiaceae", rep("Amaranthaceae", 4), "Nelumbonaceae",
            rep("Poaceae", 15), "Bromeliaceae", rep("Arecaceae", 2), "Musaceae",
            rep("Orchidaceae", 2), "Asparagaceae", "Velloziaceae",
            rep("Araceae", 2), "Zosteraceae", "Amborellaceae"
        ),
        Clade = c(
            rep("Rosids", 55), "Outgroup",
            rep("Superasterids", 23), "Outgroup",
            rep("Monocots", 26), "Outgroup"
        )
    )

    slice_sample(species_metadata, n = 10) # inspect 10 randomly sampled rows
    ##                        Species Abbrev          Family         Clade
    ## 1               Betula pendula   Bpen      Betulaceae        Rosids
    ## 2  Amaranthus hypochondriacus   Ahyp   Amaranthaceae Superasterids
    ## 3                Carica papaya    cpa      Caricaceae        Rosids
    ## 4       Pyrus x bretschneideri    pbr        Rosaceae        Rosids
    ## 5                  Ipomoea nil   Inil  Convolvulaceae Superasterids
    ## 6     Thellungiella salsuginea    tsa    Brassicaceae        Rosids
    ## 7         Tarenaya hassleriana    tha      Cleomaceae        Rosids
    ## 8      Leavenworthia alabamica    lal    Brassicaceae        Rosids
```

```
## 9        Spirodela polyrhiza    spo        Araceae      Monocots
## 10       Populus trichocarpa    ptr     Salicaceae        Rosids
```

As some species do not have an underscore (i.e., "_") after their abbreviations, we will add them.

```
#----Add underscore (i.e., "_") in front of species abbreviations in genes------
new_abbrev <- paste0(species_metadata$Abbrev, "_")
names(new_abbrev) <- paste0("^", species_metadata$Abbrev)

busco_network <- busco_network %>%
    dplyr::mutate(X1 = str_replace_all(X1, new_abbrev)) %>%
    dplyr::mutate(X1 = str_replace_all(X1, "__", "_")) %>%
    dplyr::mutate(X2 = str_replace_all(X2, new_abbrev)) %>%
    dplyr::mutate(X2 = str_replace_all(X2, "__", "_"))

head(busco_network)
## # A tibble: 6 x 2
##   X1            X2
##   <chr>         <chr>
## 1 aar_AA1G00152 bvu_Bv7_172870_irmg
## 2 aar_AA1G00152 csi_1g021926
## 3 aar_AA1G00152 csi_1g021868
## 4 aar_AA1G00152 dca_018939
## 5 aar_AA1G00152 pbr_028008.1
## 6 aar_AA1G00152 pbr_042334.1
```

Now, we will cluster the network using the Infomap algorithm and obtain phylogenomic profiles.

```
#----Cluster network and get phylogenomic profiles-----------------------------
clusters <- cluster_network(busco_network)
profiles <- phylogenomic_profile(clusters)
```

Finally, let's visualize phylogenomic profiles as a heatmap, as in Zhao and Schranz (2019). We will also create a named vector with species abbreviations and their full names, so that the full names are displayed in the rows of the heatmap.

```
# Add a column to the metadata data frame containing species names with:
# First letter of genus, whole specific epithet (e.g., Athaliana, Gmax, etc.)
species_metadata <- species_metadata %>%
    mutate(Genus = word(Species)) %>%
    mutate(Genus = str_sub(Genus, 1, 1)) %>%
    mutate(se = word(Species, 2)) %>%
    mutate(names = str_c(Genus, se)) %>%
    mutate(names = str_replace_all(names, "Px", "Pbre")) %>%
    dplyr::select(Abbrev, Name = names, Clade)

species_metadata$Name[92] <- "Zmays_v4"

slice_sample(species_metadata, n = 5) # see 5 randomly sampled rows of the data frame
##   Abbrev          Name          Clade
```

```
## 1    phe Pheterocycla      Monocots
## 2    zma        Zmays      Monocots
## 3    sin     Sindicum Superasterids
## 4    zom      Zmarina      Monocots
## 5    spe   Spennellii Superasterids

# Get species order for the heatmap
species_order <- setNames(species_metadata$Abbrev, species_metadata$Name)
head(species_order)
##   Vradiata Vangularis  Pvulgaris       Gmax     Ccajan  Tpratense
##      "vra"      "van"      "pvu"      "gma"      "cca"      "tpr"

#----Plot profiles-------------------------------------------------------------
plot_profiles(
    profiles,
    species_annotation = species_metadata[, c("Abbrev", "Clade")],
    cluster_species = species_order
)
```

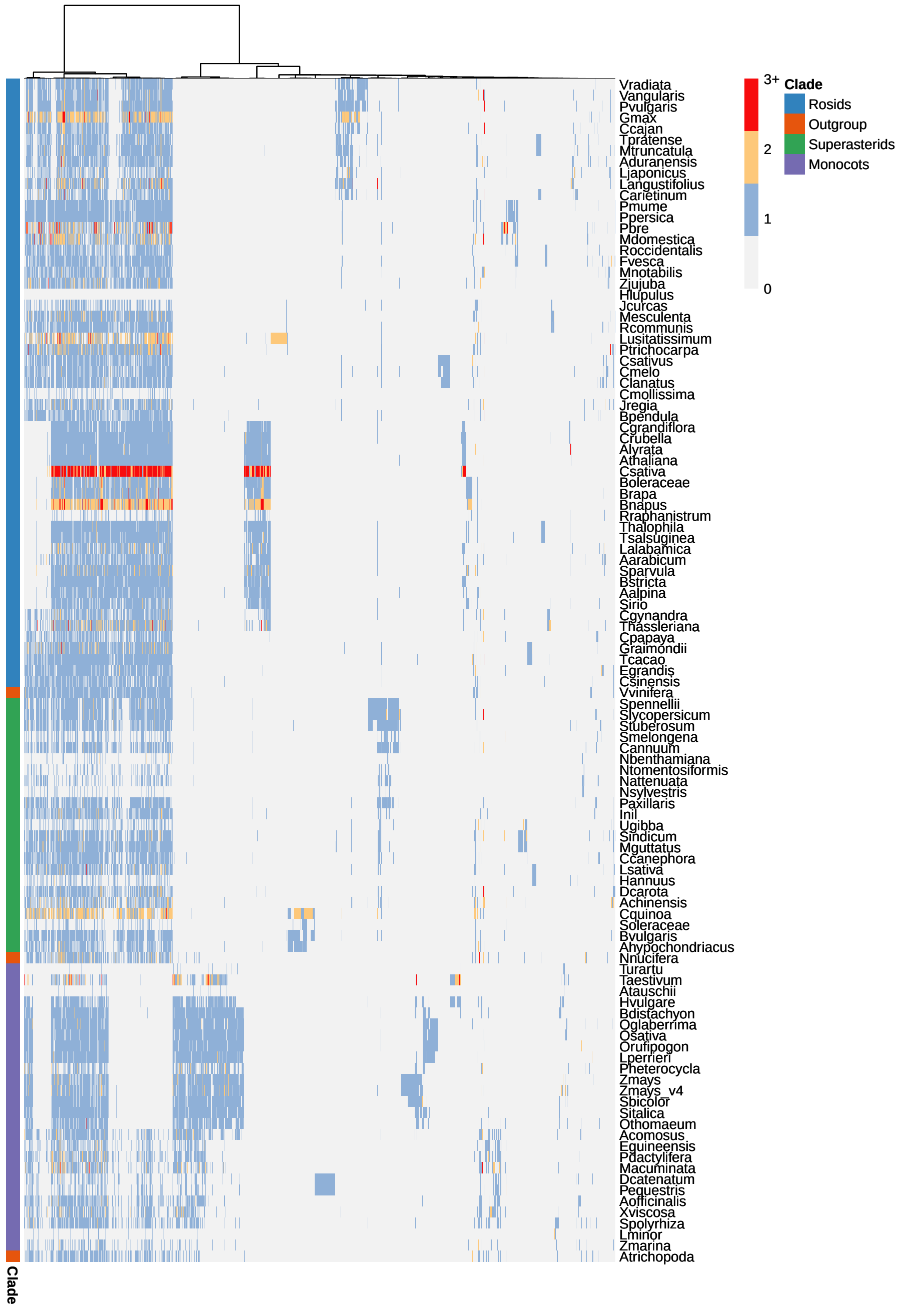

As in Figure 1B, we can see deeply conserved synteny clusters and clade-specific clusters.

Section 2: Rebuilding the angiosperm phylogeny from Zhao et al. (2021)

Here, we will infer a microsynteny-based phylogeny of angiosperms, as we did in Zhao et al. (2021). Data were obtained from the Dataverse repository associated with the original publication (DOI: 10.7910/DVN/7ZZWIH). To save time, we will not infer the network and build phylogenomic profiles again. Instead, we will use the pre-built profiles to infer the phylogeny. Our goal here is to reproduce the angiosperm phylogeny in Fig. 1D.

First, let's obtain the pre-built profiles and create a binarized and transposed profile matrix.

```
#----Get data------------------------------------------------------------------
profiles_file <- file.path(tempdir(), "profiles.tar.gz")
if(!file.exists(profiles_file)) {
    download.file(
        url = file.path(
            "https://dataverse.harvard.edu/api/access/datafile/",
            ":persistentId?persistentId=doi:10.7910/DVN/7ZZWIH/HFZBGE"
        ),
        destfile = profiles_file
    )
}
system2("tar", args = c("-zxvf", profiles_file))

#----Load file-----------------------------------------------------------------
prof <- read.table(
    "123genome_profiled_allsize", header = TRUE, sep = " ",
    row.names = 1
)
prof <- as.matrix(prof)

prof[1:10, 1:10] # inspect file
##    pmu ppe pbr Mald Rchi fve roc Dryd Tori Pand
## 1   55  62  83  144   97  52   9   31  101  123
## 2    6   6   8   12    6   6   6    6    6    5
## 3    6   6  10   13    6   7   4    6   12   10
## 4   14  13  19   13    9   8   7    9    9   10
## 5    4   4   4    7   12   8   3    3    3    3
## 6    3   3   5    6    3   3   3    2    3    3
## 7    9  10  20   15   17  17   6    5    7    7
## 8    3   3   4    7    3   2   3    4    2    3
## 9    7   7  16   14    6   6   4    6    7    4
## 10  10  10  18    8    8   4   3    5    5    3

#----Binarize and transpose profiles-------------------------------------------
transposed_profiles <- binarize_and_transpose(prof)
transposed_profiles[1:10, 1:10]
##     1 2 3 4 5 6 7 8 9 10
## pmu 1 1 1 1 1 1 1 1 1  1
## ppe 1 1 1 1 1 1 1 1 1  1
## pbr 1 1 1 1 1 1 1 1 1  1
```

```
## Mald 1 1 1 1 1 1 1 1 1  1
## Rchi 1 1 1 1 1 1 1 1 1  1
## fve  1 1 1 1 1 1 1 1 1  1
## roc  1 1 1 1 1 1 1 1 1  1
## Dryd 1 1 1 1 1 1 1 1 1  1
## Tori 1 1 1 1 1 1 1 1 1  1
## Pand 1 1 1 1 1 1 1 1 1  1
```

Now, we will use this transposed binary matrix to infer a phylogenetic tree. As an outgroup, we will use *Amborella trichopoda*, which is represented by the acronym `atr`.

```
#----Infer microsynteny-based phylogeny----------------------------------------
## Using Amborella trichopoda as an outgroup
phylo <- infer_microsynteny_phylogeny(transposed_profiles, outgroup = "atr")

## Read tree file
treefile <- list.files(tempdir(), pattern = ".treefile", full.names = TRUE)
angiosperm_phylogeny <- treeio::read.tree(treefile)
```

Finally, we can plot the phylogeny using the Bioconductor package *ggtree*. To make the plot look nicer, we will replace species acronyms with their full names.

```
# Replace abbreviations with full names
angiosperm_phylogeny$tip.label
##   [1] "pmu"  "ppe"  "pbr"  "Mald" "Rchi" "fve"  "roc"  "Dryd" "Tori" "Pand"
##  [11] "Mnot" "Zjuj" "csa"  "cme"  "cla"  "Cuma" "Datg" "Begf" "vra"  "van"
##  [21] "pvu"  "gma"  "cca"  "tpr"  "mtr"  "car"  "lja"  "Anan" "Lang" "adu"
##  [31] "Bpen" "Cgla" "Cill" "Qrob" "cru"  "Csat" "Alyr" "ath"  "Bost" "Lmey"
##  [41] "bol"  "bnp"  "bra"  "spa"  "thh"  "tsa"  "Alp"  "aar"  "cgy"  "tha"
##  [51] "cpa"  "Goba" "Ghir" "gra"  "Dzib" "tca"  "Cmax" "csi"  "Xsor" "mes"
##  [61] "rco"  "ptr"  "lus"  "egr"  "Pgra" "spe"  "sly"  "stu"  "Caba" "Cach"
##  [71] "can"  "pax"  "Inil" "Cuca" "coc"  "sin"  "mgu"  "Oeur" "Lsat" "HanX"
##  [81] "dca"  "ach"  "Aeri" "Cqui" "bvu"  "Ahyp" "Mole" "Kalf" "vvi"  "nnu"
##  [91] "Psom" "Mcor" "Aqco" "sbi"  "Sacc" "Zmay" "sit"  "Sevi" "Ecru" "oth"
## [101] "ogl"  "osa"  "oru"  "Opun" "lpe"  "Trdc" "HORV" "bdi"  "aco"  "mac"
## [111] "egu"  "Pdac" "peq"  "Ashe" "Aoff" "Xvis" "spo"  "zom"  "Peam" "CKAN"
## [121] "Lchi" "atr"  "Nymp"
angiosperm_phylogeny$tip.label <- stringr::str_replace_all(
    angiosperm_phylogeny$tip.label,
    c("^pmu" = "Prunus mume",
      "^ppe" = "Prunus persica",
      "^pbr" = "Pyrus x bretschneideri",
      "^Mald" = "Malus domestica",
      "^Rchi" = "Rosa chinensis",
      "^fve" = "Fragaria vesca",
      "^roc" = "Rubus occidentalis",
      "^Dryd" = "Dryas drummondii",
      "^Pand" = "Parasponia andersonii",
      "^Tori" = "Trema orientale",
      "^Mnot" = "Morus notabilis",
      "^Zjuj" = "Ziziphus jujuba",
      "^cme" = "Cucumis melo",
```

```
"^csa" = "Cucumis sativus",
"^cla" = "Citrullus lanatus",
"^Cuma" = "Cucurbita maxima",
"^Begf" = "Begonia fuchsioides",
"^Datg" = "Datisca glomerata",
"^van" = "Vigna angularis",
"^vra" = "Vigna radiata",
"^pvu" = "Phaseolus vulgaris",
"^gma" = "Glycine max",
"^cca" = "Cajanus cajan",
"^mtr" = "Medicago truncatula",
"^tpr" = "Trifolium pratense",
"^car" = "Cicer arietinum",
"^lja" = "Lotus japonicus",
"^Lang" = "Lupinus angustifolius",
"^Anan" = "Ammopiptanthus nanus",
"^adu" = "Arachis duranensis",
"^Cgla" = "Casuarina glauca",
"^Bpen" = "Betula pendula",
"^Cill" = "Carya illinoinensis",
"^Qrob" = "Quercus robur",
"^bnp" = "Brassica napus",
"^bol" = "Brassica oleracea",
"^bra" = "Brassica rapa",
"^spa" = "Schrenkiella parvula",
"^tsa" = "Thellungiella salsuginea",
"^thh" = "Thellungiella halophila",
"^ath" = "Arabidopsis thaliana",
"^Alyr" = "Arabidopsis lyrata",
"^Csat" = "Camelina sativa",
"^cru" = "Capsella rubella",
"^Bost" = "Boechera stricta",
"^Lmey" = "Lepidium meyenii",
"^Alp" = "Arabis alpina",
"^aar" = "Aethionema arabicum",
"^tha" = "Tarenaya hassleriana",
"^cgy" = "Cleome gynandra",
"^cpa" = "Carica papaya",
"^Ghir" = "Gossypium hirsutum",
"^Goba" = "Gossypium barbadense",
"^gra" = "Gossypium raimondii",
"^Dzib" = "Durio zibethinus",
"^tca" = "Theobroma cacao",
"^csi" = "Citrus sinensis",
"^Cmax" = "Citrus maxima",
"^Xsor" = "Xanthoceras sorbifolium",
"^rco" = "Ricinus communis",
"^mes" = "Manihot esculenta",
"^ptr" = "Populus trichocarpa",
"^lus" = "Linum usitatissimum",
"^Pgra" = "Punica granatum",
```

```
               "^egr" = "Eucalyptus grandis",
               "^Cach" = "Capsicum chinense",
               "^Caba" = "Capsicum baccatum",
               "^can" = "Capsicum annuum",
               "^sly" = "Solanum lycopersicum",
               "^spe" = "Solanum pennellii",
               "^stu" = "Solanum tuberosum",
               "^pax" = "Petunia axillaris",
               "^Cuca" = "Cuscuta campestris",
               "^Inil" = "Ipomoea nil",
               "^coc" = "Coffea canephora",
               "^mgu" = "Mimulus guttatus",
               "^sin" = "Sesamum indicum",
               "^Oeur" = "Olea europaea",
               "^HanX" = "Helianthus annuus",
               "^Lsat" = "Lactuca sativa",
               "^dca" = "Daucus carota",
               "^Aeri" = "Actinidia eriantha",
               "^ach" = "Actinidia chinensis",
               "^bvu" = "Beta vulgaris",
               "^Cqui" = "Chenopodium quinoa",
               "^Ahyp" = "Amaranthus hypochondriacus",
               "^Kalf" = "Kalanchoe fedtschenkoi",
               "^Mole" = "Malania oleifera",
               "^vvi" = "Vitis vinifera",
               "^nnu" = "Nelumbo nucifera",
               "^Mcor" = "Macleaya cordata",
               "^Psom" = "Papaver somniferum",
               "^Aqco" = "Aquilegia coerulea",
               "^Sevi" = "Setaria viridis",
               "^sit" = "Setaria italica",
               "^Ecru" = "Echinochloa crus-galli",
               "^Sacc" = "Saccharum officinarum",
               "^sbi" = "Sorghum bicolor",
               "^Zmay" = "Zea mays",
               "^oth" = "Oropetium thomaeum",
               "^osa" = "Oryza sativa",
               "^ogl" = "Oryza glaberrima",
               "^oru" = "Oryza rufipogon",
               "^Opun" = "Oryza punctata",
               "^lpe" = "Leersia perrieri",
               "^HORV" = "Hordeum vulgare",
               "^Trdc" = "Triticum turgidum",
               "^bdi" = "Brachypodium distachyon",
               "^aco" = "Ananas comosus",
               "^mac" = "Musa acuminata",
               "^Pdac" = "Phoenix dactylifera",
               "^egu" = "Elaeis guineensis",
               "^Ashe" = "Apostasia shenzhenica",
               "^peq" = "Phalaenopsis equestris",
               "^Aoff" = "Asparagus officinalis",
```

```
            "^Xvis" = "Xerophyta viscosa",
            "^zom" = "Zostera marina",
            "^spo" = "Spirodela polyrhiza",
            "^CKAN" = "Cinnamomum kanehirae",
            "^Peam" = "Persea americana",
            "^Lchi" = "Liriodendron chinense",
            "^Nymp" = "Nymphaea colorata",
            "^atr" = "Amborella trichopoda"
            )
      )

      #----Plot tree------------------------------------------------------------------
      suppressPackageStartupMessages(library(ggtree))
      ggtree(angiosperm_phylogeny) +
          geom_tiplab(size = 3) +
          xlim(0, 0.3)
```

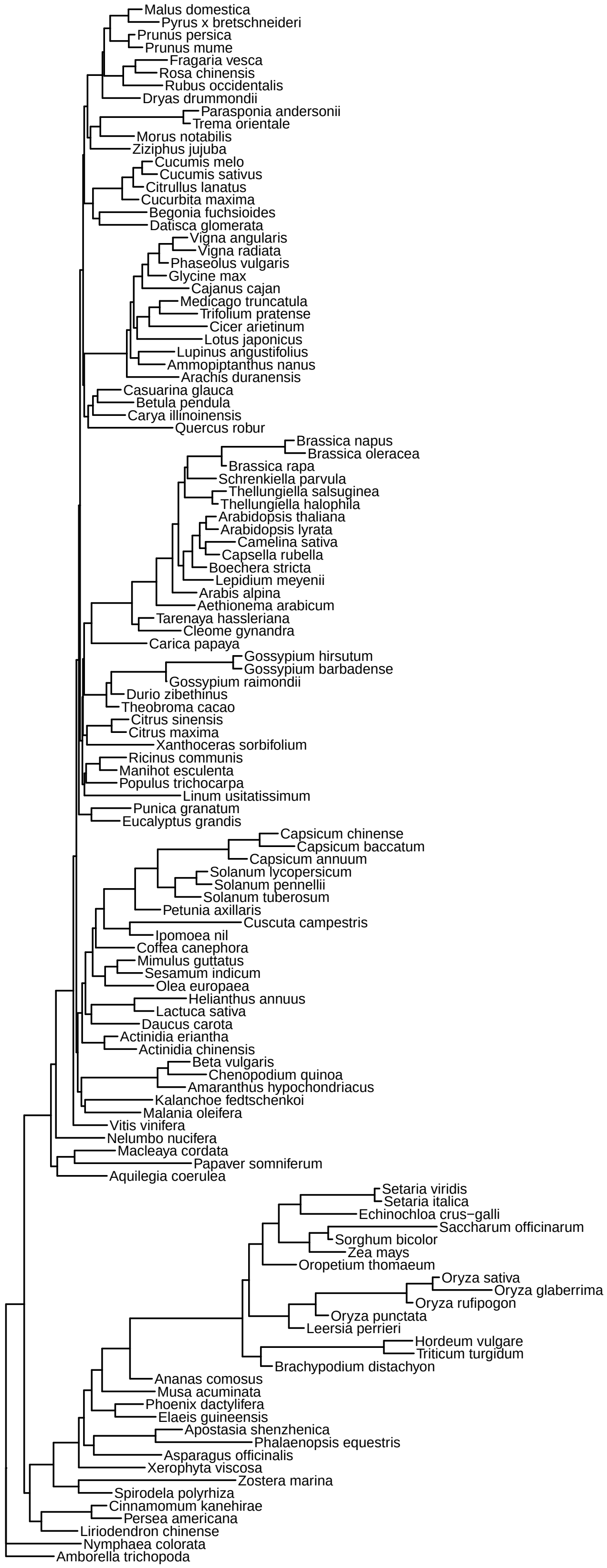
Malus domestica
Pyrus x bretschneideri
Prunus persica
Prunus mume
Fragaria vesca
Rosa chinensis
Rubus occidentalis
Dryas drummondii
Parasponia andersonii
Trema orientale
Morus notabilis
Ziziphus jujuba
Cucumis melo
Cucumis sativus
Citrullus lanatus
Cucurbita maxima
Begonia fuchsioides
Datisca glomerata
Vigna angularis
Vigna radiata
Phaseolus vulgaris
Glycine max
Cajanus cajan
Medicago truncatula
Trifolium pratense
Cicer arietinum
Lotus japonicus
Lupinus angustifolius
Ammopiptanthus nanus
Arachis duranensis
Casuarina glauca
Betula pendula
Carya illinoinensis
Quercus robur
Brassica napus
Brassica oleracea
Brassica rapa
Schrenkiella parvula
Thellungiella salsuginea
Thellungiella halophila
Arabidopsis thaliana
Arabidopsis lyrata
Camelina sativa
Capsella rubella
Boechera stricta
Lepidium meyenii
Arabis alpina
Aethionema arabicum
Tarenaya hassleriana
Cleome gynandra
Carica papaya
Gossypium hirsutum
Gossypium barbadense
Gossypium raimondii
Durio zibethinus
Theobroma cacao
Citrus sinensis
Citrus maxima
Xanthoceras sorbifolium
Ricinus communis
Manihot esculenta
Populus trichocarpa
Linum usitatissimum
Punica granatum
Eucalyptus grandis
Capsicum chinense
Capsicum baccatum
Capsicum annuum
Solanum lycopersicum
Solanum pennellii
Solanum tuberosum
Petunia axillaris
Cuscuta campestris
Ipomoea nil
Coffea canephora
Mimulus guttatus
Sesamum indicum
Olea europaea
Helianthus annuus
Lactuca sativa
Daucus carota
Actinidia eriantha
Actinidia chinensis
Beta vulgaris
Chenopodium quinoa
Amaranthus hypochondriacus
Kalanchoe fedtschenkoi
Malania oleifera
Vitis vinifera
Nelumbo nucifera
Macleaya cordata
Papaver somniferum
Aquilegia coerulea
Setaria viridis
Setaria italica
Echinochloa crus−galli
Saccharum officinarum
Sorghum bicolor
Zea mays
Oropetium thomaeum
Oryza sativa
Oryza glaberrima
Oryza rufipogon
Oryza punctata
Leersia perrieri
Hordeum vulgare
Triticum turgidum
Brachypodium distachyon
Ananas comosus
Musa acuminata
Phoenix dactylifera
Elaeis guineensis
Apostasia shenzhenica
Phalaenopsis equestris
Asparagus officinalis
Xerophyta viscosa
Zostera marina
Spirodela polyrhiza
Cinnamomum kanehirae
Persea americana
Liriodendron chinense
Nymphaea colorata
Amborella trichopoda

Section 3: Exploring synteny networks for Chlorophyta genomes

In this final section, we will demonstrate the whole pipeline using the genomes of algae from the Chlorophyta clade.

First, let load the data on-the-fly from Pico-Plaza 3.0 (Van Bel et al. 2018).

```
species <- c(
    Aprotothecoides = "apr",
    Helicosporidiumsp = "hsp",
    Chlorellasp = "cnc64a",
    PicRCC4223 = "prcc4223",
    PicSE3 = "pse3",
    Asterochlorissp = "acg",
    Csubellipsoidea = "cvu",
    Creinhardtii = "cre",
    Vcarteri = "vca",
    Bprasinos = "bprrcc1105",
    Otauri = "ota",
    Osp = "orcc809",
    Olucimarinus = "olu",
    Omediterraneus = "ome",
    Msp = "mrcc299",
    Mpusilla = "mpu",
    Ppatens = "ppa"
)

#----Get proteomes----------------------------------------------------------------
proteome_urls <- file.path(
    "ftp://ftp.psb.ugent.be/pub/plaza/plaza_pico_03/Fasta",
    paste0("proteome.selected_transcript.", species, ".fasta.gz")
)
names(proteome_urls) <- names(species)

## Headers in .fa files have protein IDs. Read proteomes and keep only gene IDs
proteomes <- lapply(proteome_urls, function(x) {
    seq <- Biostrings::readAAStringSet(x)
    names(seq) <- gsub(".* \\| ", "", names(seq))
    return(seq)
})

#----Get gene ranges--------------------------------------------------------------
granges_urls <- file.path(
    "ftp://ftp.psb.ugent.be/pub/plaza/plaza_pico_03/GFF", species,
    paste0("annotation.selected_transcript.exon_features.", species, ".gff3.gz")
)
names(granges_urls) <- names(species)

## Read files and keep only required features and columns
annotation <- lapply(granges_urls, function(x) {
```

```
    dfile <- file.path(tempdir(), basename(x))
    utils::download.file(x, dfile)
    ranges <- rtracklayer::import(dfile)
    unlink(dfile)

    ranges <- ranges[ranges$type == "gene", ]
    ranges$Parent <- NULL
    ranges$phase <- NULL
    ranges$score <- NULL
    ranges$source <- NULL
    ranges$pid <- NULL
    return(ranges)
})
```

Now, let's infer the synteny network.

```
#----Data processing-------------------------------------------------------------
## Check if input objects satisfy required conditions to enter the pipeline
check_input(proteomes, annotation)

## Process the data
pdata <- process_input(proteomes, annotation)

#----Infer synteny network-------------------------------------------------------
## Run DIAMOND
diamond <- run_diamond(seq = pdata$seq)

## Network inference per se
algae_network <- infer_syntenet(
    blast_list = diamond,
    annotation = pdata$annotation
)
```

Now, we have the synteny network for Chlorophyta algae. Let's see what it looks like.

```
slice_sample(algae_network, n = 10) # inspect 10 randomly selected rows
##                       Anchor1               Anchor2
## 1      Osp_ORCC809_14G01330      Otau_OT_15G01000
## 2  Bpra_BPRRCC1105_07G04710      Omed_OM_15G02500
## 3           Oluc_OL02G05400      Omed_OM_04G02960
## 4           Oluc_OL16G02530  Osp_ORCC809_16G00160
## 5           Oluc_OL16G01300      Otau_OT_17G01220
## 6           Crei_CR06G00520       Vcar_VC00G40860
## 7          Omed_OM_10G02570  Osp_ORCC809_09G03500
## 8           Mpus_MP16G00270       Oluc_OL16G00220
## 9     Chlo_CNC64A_003G03260 PicR_RCC4223.03g02610
## 10          Aste_AC00G40930       Csub_CV00G35230
```

Next, we will cluster the network and explore phylogenomic profiles.

```
#----Network clustering and phylogenomic profiling-------------------------------
## Get synteny clusters
```

```
clusters <- cluster_network(algae_network)
head(clusters) # inspect clusters
##                    Gene Cluster
## 1 Chlo_CNC64A_028G00030       1
## 2 Chlo_CNC64A_028G00040       2
## 3 Chlo_CNC64A_028G00070       3
## 4 Chlo_CNC64A_028G00080       4
## 5 Chlo_CNC64A_028G00110       5
## 6 Chlo_CNC64A_028G00140       6

## Get phylogenomic profiles
profiles <- phylogenomic_profile(clusters)
head(profiles) # inspect the profile matrix
##
##     Apro Aste Bpra Chlo Crei Csub Mpus Msp Oluc Omed Osp Otau PicR PicS Ppat
##   1    1    1    0    5   21    5    0   0    0    0   0    0    0    0    0
##   2    1    1    0    4   22    5    0   0    0    0   0    0    0    0    0
##   3    0    1    0    4   21    6    0   0    0    0   0    0    0    0    0
##   4    0    0    0    2    0    0    0   0    0    0   0    0    0    0    0
##   5    0    0    0    6    2    0    0   0    0    0   0    0    0    0    0
##   6    0    0    0    2    0    0    0   0    0    0   0    0    0    0    0
##
##     Vcar
##   1    4
##   2    5
##   3    4
##   4    0
##   5    0
##   6    0
```

We can then plot these phylogenomic profiles as a heatmap.

```
#----Plot phylogenomic profiles-------------------------------------------------
## Create a data frame of species metadata
species_meta <- data.frame(
    Species = c(
        "Aprotothecoides", "Chlorellasp", "PicRCC4223",
        "PicSE3", "Asterochlorissp", "Csubellipsoidea", "Creinhardtii",
        "Vcarteri", "Bprasinos", "Otauri", "Osp", "Olucimarinus",
        "Omediterraneus", "Msp", "Mpusilla", "Ppatens"
    ),
    Abbrev = c(
        "Apro", "Chlo", "PicR", "PicS", "Aste", "Csub", "Crei",
        "Vcar", "Bpra", "Otau", "Osp", "Oluc", "Omed", "Msp",
        "Mpus", "Ppat"
    ),
    Clade = c(
        rep("Trebouxiophyceae", 6),
        rep("Chlamydomonadales", 2),
        rep("Marmiellales", 7),
        "Outgroup"
```

```
        )
    )

    ## Create a vector of species order
    species_order <- setNames(species_meta$Abbrev, species_meta$Species)

    ## Plotting heatmap
    p_prof <- plot_profiles(
        profiles,
        species_annotation = species_meta[, c("Abbrev", "Clade")],
        cluster_species = species_order
    )
```

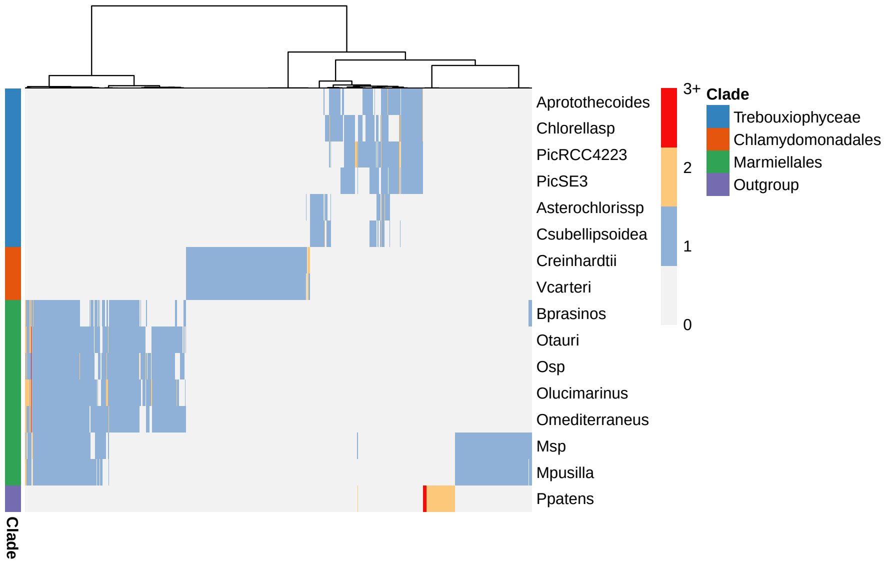

We can see that the phylogenomic profiles of synteny clusters match the species' phylogeny. For instance, there are Chlamydomonadales-specific clusters, Marmiellales-specific clusters, and Trebouxiophyceae-specific clusters. Besides, the phylogenomic profiles of algae is very different from that of *Physcomitrium patens*.

Finally, let's use these profiles to infer a microsynteny-based phylogeny. We will use *Physcomitrium patens* as outgroup for the tree. Here, as the phylogenomic profiles do not contain constant sites, we will add `+ASC` to the model to perform an ascertainment bias correction.

```
    #----Infer microsynteny-based phylogeny--------------------------------------
    ## Binarize and tranpose profiles matrix
    bt_mat <- binarize_and_transpose(profiles)
    bt_mat[1:5, 1:5] # inspect data
    ##
    ##        1 2 3 4 5
    ##   Apro 1 1 0 0 0
    ##   Aste 1 1 1 0 0
    ##   Bpra 0 0 0 0 0
    ##   Chlo 1 1 1 1 1
```

```
##   Crei 1 1 1 0 1

## Infer phylogeny using P. patens as outgroup
phylo <- infer_microsynteny_phylogeny(
    bt_mat,
    outgroup = "Ppat",
    model = "MK+ASC+R",
    threads = 1
)

## Plot tree
suppressPackageStartupMessages(library(ggtree))
algae_tree <- treeio::read.tree(phylo[10])
ggtree(algae_tree) +
    geom_tiplab(size = 3) +
    xlim(0, 0.2)
```

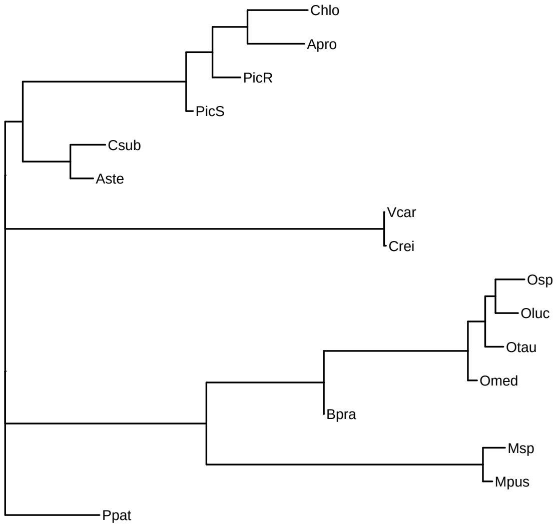

Overall, the microsynteny-based tree is in line with the alignment-based tree (see https://bioinformatics.psb.ugent.be/plaza/versions/plaza_pico_03/). However, the tree topology shows Picochlorum_RCC4223 closer to the Chlorella sp-Auxenochlorella protothecoides clade than to Picochlorum sp. SENEW3 (SE3). The phylogenetic relationships could be better resolved if more genomes are included.

Benchmarking runtime for increasingly large data sets

To infer synteny networks, *syntenet* relies on pairwise similarity searches with DIAMOND, whose output is used as input to synteny detection with a native version of the MCScanX algorithm. For N species, N^2 DIAMOND searches will be performed, and $N(N+1)/2$ synteny detection runs.

To provide readers with a more detailed summary of how synteny network inference scales with bigger data sets, we will infer synteny networks using increasingly larger subsets of the Chlorophyta data set. We will start with 2 species, and then we will add 2 species in every run, until we reach the complete data set (N = 16 species).

```
# Run pairwise DIAMOND searches for increasingly larger subsets of the data
outd <- file.path(tempdir(), paste0("d", 1:8))

time_diamond <- microbenchmark::microbenchmark(
    d1 <- run_diamond(seq = pdata$seq[seq(1, 2)], outdir = outd[1]),
    d2 <- run_diamond(seq = pdata$seq[seq(1, 4)], outdir = outd[2]),
    d3 <- run_diamond(seq = pdata$seq[seq(1, 6)], outdir = outd[3]),
    d4 <- run_diamond(seq = pdata$seq[seq(1, 8)], outdir = outd[4]),
    d5 <- run_diamond(seq = pdata$seq[seq(1, 10)], outdir = outd[5]),
    d6 <- run_diamond(seq = pdata$seq[seq(1, 12)], outdir = outd[6]),
    d7 <- run_diamond(seq = pdata$seq[seq(1, 14)], outdir = outd[7]),
    d8 <- run_diamond(seq = pdata$seq[seq(1, 16)], outdir = outd[8]),
    times = 1
)

# Infer a synteny network for each subset
outn <- file.path(tempdir(), paste0("net", 1:8))

time_syntenet <- microbenchmark::microbenchmark(
    n1 <- infer_syntenet(d1, pdata$annotation[seq(1, 2)], outdir = outn[1]),
    n2 <- infer_syntenet(d2, pdata$annotation[seq(1, 4)], outdir = outn[2]),
    n3 <- infer_syntenet(d3, pdata$annotation[seq(1, 6)], outdir = outn[3]),
    n4 <- infer_syntenet(d4, pdata$annotation[seq(1, 8)], outdir = outn[4]),
    n5 <- infer_syntenet(d5, pdata$annotation[seq(1, 10)], outdir = outn[5]),
    n6 <- infer_syntenet(d6, pdata$annotation[seq(1, 12)], outdir = outn[6]),
    n7 <- infer_syntenet(d7, pdata$annotation[seq(1, 14)], outdir = outn[7]),
    n8 <- infer_syntenet(d8, pdata$annotation[seq(1, 16)], outdir = outn[8]),
    times = 1
)
```

Now, let's visually explore runtime for the DIAMOND searches, synteny network inferences, and a combination of both.

```
# Now, combining runtime results in a single, tidy data frame
runtime_functions <- bind_rows(
    # run_diamond() runtime
    as.data.frame(time_diamond) %>%
        arrange(time) %>%
        mutate(
            Data = paste("Subset", 1:8),
            Seconds = time / 10^9,
            Function = "run_diamond()"
        ) %>%
        dplyr::select(Data, Seconds, Function),
    # infer_syntenet() runtime
    as.data.frame(time_syntenet) %>%
        arrange(time) %>%
```

```
        mutate(
            Data = paste("Subset", 1:8),
            Seconds = time / 10^9,
            Function = "infer_syntenet()"
        ) %>%
        dplyr::select(Data, Seconds, Function)
)

runtime_all <- runtime_functions %>%
    group_by(Data) %>%
    summarise(Seconds = sum(Seconds)) %>%
    mutate(Function = "run_diamond() + infer_syntenet()") %>%
    bind_rows(runtime_functions) %>%
    mutate(Data = factor(Data, levels = paste("Subset", 8:1))) %>%
    as.data.frame()

runtime_all
##        Data     Seconds                         Function
## 1  Subset 1   7.5593162 run_diamond() + infer_syntenet()
## 2  Subset 2  26.6445645 run_diamond() + infer_syntenet()
## 3  Subset 3  58.3822717 run_diamond() + infer_syntenet()
## 4  Subset 4 138.3175053 run_diamond() + infer_syntenet()
## 5  Subset 5 226.2897421 run_diamond() + infer_syntenet()
## 6  Subset 6 284.8873378 run_diamond() + infer_syntenet()
## 7  Subset 7 370.7832199 run_diamond() + infer_syntenet()
## 8  Subset 8 484.3175754 run_diamond() + infer_syntenet()
## 9  Subset 1   7.2006111                    run_diamond()
## 10 Subset 2  24.0096182                    run_diamond()
## 11 Subset 3  54.8228134                    run_diamond()
## 12 Subset 4 128.3520661                    run_diamond()
## 13 Subset 5 210.3434089                    run_diamond()
## 14 Subset 6 263.6309493                    run_diamond()
## 15 Subset 7 343.2867030                    run_diamond()
## 16 Subset 8 455.3109649                    run_diamond()
## 17 Subset 1   0.3587051                 infer_syntenet()
## 18 Subset 2   2.6349463                 infer_syntenet()
## 19 Subset 3   3.5594583                 infer_syntenet()
## 20 Subset 4   9.9654392                 infer_syntenet()
## 21 Subset 5  15.9463332                 infer_syntenet()
## 22 Subset 6  21.2563886                 infer_syntenet()
## 23 Subset 7  27.4965169                 infer_syntenet()
## 24 Subset 8  29.0066105                 infer_syntenet()

# Plot runtime results
runtime_plot <- ggplot(runtime_all, aes(x = Seconds, y = Data)) +
    geom_point(aes(color = Function), show.legend = FALSE) +
    ggsci::scale_color_aaas() +
    facet_wrap(~Function, scales = "free_x") +
    labs(
        x = "Time (seconds)", y = "",
        title = "Runtime of syntenet functions for increasingly large data sets",
```

```
            subtitle = "Chlorophyta data set; subset 1 = 2 species, subset 8 = 16 species"
        ) +
        theme_bw()

runtime_plot
```

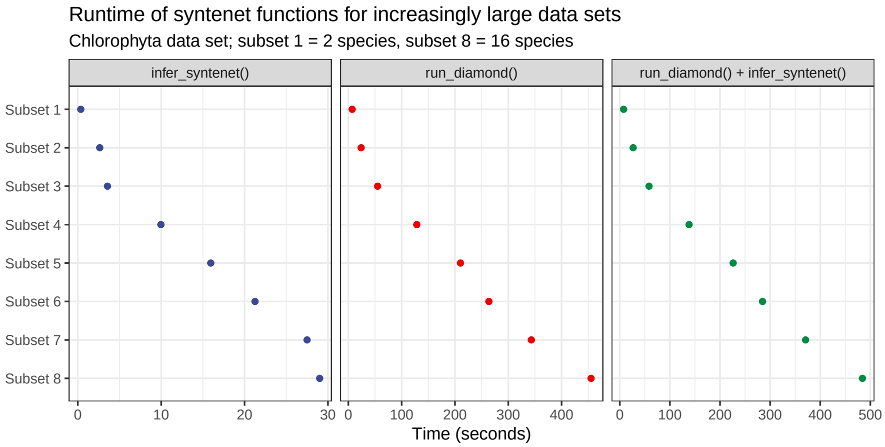

The benchmark demonstrates that the entire process of inferring a synteny network can take up to 484 seconds (~8 minutes) for the full Chlorophyta data set (N = 16 species), but most of the time spent is due to DIAMOND similarity searches (455 seconds, 94% of the total runtime). Our ported version of the MCScanX algorithm is very fast, and the `infer_syntenet()` function (responsible for synteny detection + edge list representation of the resulting anchor pairs) takes less than 30 seconds for the full Chlorophyta data set. In the future, if a faster sequence similarity search tool is developed, it could be used to reduce the total runtime of the synteny network inference.

Session information

This document was created under the following conditions.

```
## - Session info ---------------------------------------------------------------
##  setting  value
##  version  R version 4.2.1 (2022-06-23)
##  os       Ubuntu 20.04.4 LTS
##  system   x86_64, linux-gnu
##  ui       X11
##  language (EN)
##  collate  en_US.UTF-8
##  ctype    en_US.UTF-8
##  tz       Europe/Brussels
##  date     2022-11-18
##  pandoc   2.19.2 @ /usr/lib/rstudio/bin/quarto/bin/tools/ (via rmarkdown)
##
## - Packages -------------------------------------------------------------------
##  package              * version   date (UTC) lib source
##  ape                    5.6-2     2022-03-02 [1] CRAN (R 4.2.0)
```

```
## aplot                0.1.8     2022-10-09 [1] CRAN (R 4.2.1)
## assertthat           0.2.1     2019-03-21 [1] CRAN (R 4.2.0)
## backports            1.4.1     2021-12-13 [1] CRAN (R 4.2.0)
## Biobase              2.56.0    2022-04-26 [1] Bioconductor
## BiocGenerics         0.42.0    2022-04-26 [1] Bioconductor
## BiocIO               1.6.0     2022-04-26 [1] Bioconductor
## BiocManager          1.30.18   2022-05-18 [1] CRAN (R 4.2.0)
## BiocParallel         1.30.4    2022-10-11 [1] Bioconductor
## BiocStyle          * 2.25.0    2022-06-15 [1] Github (Bioconductor/BiocStyle@7150c28)
## Biostrings           2.64.1    2022-08-18 [1] Bioconductor
## bit                  4.0.4     2020-08-04 [1] CRAN (R 4.2.0)
## bit64                4.0.5     2020-08-30 [1] CRAN (R 4.2.0)
## bitops               1.0-7     2021-04-24 [1] CRAN (R 4.2.0)
## bookdown             0.29      2022-09-12 [1] CRAN (R 4.2.1)
## broom                1.0.1     2022-08-29 [1] CRAN (R 4.2.1)
## cellranger           1.1.0     2016-07-27 [1] CRAN (R 4.2.0)
## cli                  3.4.1     2022-09-23 [1] CRAN (R 4.2.1)
## coda                 0.19-4    2020-09-30 [1] CRAN (R 4.2.0)
## codetools            0.2-18    2020-11-04 [1] CRAN (R 4.2.0)
## colorspace           2.0-3     2022-02-21 [1] CRAN (R 4.2.0)
## crayon               1.5.2     2022-09-29 [1] CRAN (R 4.2.1)
## DBI                  1.1.3     2022-06-18 [1] CRAN (R 4.2.0)
## dbplyr               2.2.1     2022-06-27 [1] CRAN (R 4.2.1)
## DelayedArray         0.22.0    2022-04-26 [1] Bioconductor
## digest               0.6.29    2021-12-01 [1] CRAN (R 4.2.0)
## dplyr              * 1.0.10    2022-09-01 [1] CRAN (R 4.2.1)
## ellipsis             0.3.2     2021-04-29 [1] CRAN (R 4.2.0)
## evaluate             0.17      2022-10-07 [1] CRAN (R 4.2.1)
## fansi                1.0.3     2022-03-24 [1] CRAN (R 4.2.0)
## farver               2.1.1     2022-07-06 [1] CRAN (R 4.2.1)
## fastmap              1.1.0     2021-01-25 [1] CRAN (R 4.2.0)
## forcats            * 0.5.2     2022-08-19 [1] CRAN (R 4.2.1)
## fs                   1.5.2     2021-12-08 [1] CRAN (R 4.2.0)
## gargle               1.2.1     2022-09-08 [1] CRAN (R 4.2.1)
## generics             0.1.3     2022-07-05 [1] CRAN (R 4.2.1)
## GenomeInfoDb         1.32.4    2022-09-06 [1] Bioconductor
## GenomeInfoDbData     1.2.8     2022-05-06 [1] Bioconductor
## GenomicAlignments    1.32.1    2022-07-24 [1] Bioconductor
## GenomicRanges        1.48.0    2022-04-26 [1] Bioconductor
## ggfun                0.0.8     2022-11-07 [1] CRAN (R 4.2.1)
## ggnetwork            0.5.10    2021-07-06 [1] CRAN (R 4.2.0)
## ggplot2            * 3.4.0     2022-11-04 [1] CRAN (R 4.2.1)
## ggplotify            0.1.0     2021-09-02 [1] CRAN (R 4.2.0)
## ggsci                2.9       2018-05-14 [1] CRAN (R 4.2.0)
## ggtree             * 3.7.1.001 2022-11-10 [1] Github (YuLab-SMU/ggtree@b7ef83e)
## glue                 1.6.2     2022-02-24 [1] CRAN (R 4.2.0)
## googledrive          2.0.0     2021-07-08 [1] CRAN (R 4.2.0)
## googlesheets4        1.0.1     2022-08-13 [1] CRAN (R 4.2.1)
## gridGraphics         0.5-1     2020-12-13 [1] CRAN (R 4.2.0)
## gtable               0.3.1     2022-09-01 [1] CRAN (R 4.2.1)
## haven                2.5.1     2022-08-22 [1] CRAN (R 4.2.1)
```

```
## here              1.0.1     2020-12-13 [1] CRAN (R 4.2.0)
## hms               1.1.2     2022-08-19 [1] CRAN (R 4.2.1)
## htmltools         0.5.3     2022-07-18 [1] CRAN (R 4.2.1)
## htmlwidgets       1.5.4     2021-09-08 [1] CRAN (R 4.2.0)
## httr              1.4.4     2022-08-17 [1] CRAN (R 4.2.1)
## igraph            1.3.5     2022-09-22 [1] CRAN (R 4.2.1)
## intergraph        2.0-2     2016-12-05 [1] CRAN (R 4.2.0)
## IRanges           2.30.1    2022-08-18 [1] Bioconductor
## jsonlite          1.8.3     2022-10-21 [1] CRAN (R 4.2.1)
## knitr             1.40      2022-08-24 [1] CRAN (R 4.2.1)
## labeling          0.4.2     2020-10-20 [1] CRAN (R 4.2.0)
## lattice           0.20-45   2021-09-22 [1] CRAN (R 4.2.0)
## lazyeval          0.2.2     2019-03-15 [1] CRAN (R 4.2.0)
## lifecycle         1.0.3     2022-10-07 [1] CRAN (R 4.2.1)
## lubridate         1.8.0     2021-10-07 [1] CRAN (R 4.2.0)
## magrittr          2.0.3     2022-03-30 [1] CRAN (R 4.2.0)
## Matrix            1.5-1     2022-09-13 [1] CRAN (R 4.2.1)
## MatrixGenerics    1.8.1     2022-06-26 [1] Bioconductor
## matrixStats       0.62.0    2022-04-19 [1] CRAN (R 4.2.0)
## modelr            0.1.9     2022-08-19 [1] CRAN (R 4.2.1)
## munsell           0.5.0     2018-06-12 [1] CRAN (R 4.2.0)
## network           1.18.0    2022-10-06 [1] CRAN (R 4.2.1)
## networkD3         0.4       2017-03-18 [1] CRAN (R 4.2.0)
## nlme              3.1-160   2022-10-10 [1] CRAN (R 4.2.1)
## patchwork         1.1.2     2022-08-19 [1] CRAN (R 4.2.1)
## pheatmap          1.0.12    2019-01-04 [1] CRAN (R 4.2.0)
## pillar            1.8.1     2022-08-19 [1] CRAN (R 4.2.1)
## pkgconfig         2.0.3     2019-09-22 [1] CRAN (R 4.2.0)
## purrr           * 0.3.5     2022-10-06 [1] CRAN (R 4.2.1)
## R6                2.5.1     2021-08-19 [1] CRAN (R 4.2.0)
## Rclusterpp        0.2.6     2022-11-18 [1] Github (nolanlab/Rclusterpp@a073806)
## RColorBrewer      1.1-3     2022-04-03 [1] CRAN (R 4.2.0)
## Rcpp              1.0.9     2022-07-08 [1] CRAN (R 4.2.1)
## RCurl             1.98-1.9  2022-10-03 [1] CRAN (R 4.2.1)
## readr           * 2.1.3     2022-10-01 [1] CRAN (R 4.2.1)
## readxl            1.4.1     2022-08-17 [1] CRAN (R 4.2.1)
## reprex            2.0.2     2022-08-17 [1] CRAN (R 4.2.1)
## restfulr          0.0.15    2022-06-16 [1] CRAN (R 4.2.0)
## rjson             0.2.21    2022-01-09 [1] CRAN (R 4.2.0)
## rlang             1.0.6     2022-09-24 [1] CRAN (R 4.2.1)
## rmarkdown         2.17      2022-10-07 [1] CRAN (R 4.2.1)
## rprojroot         2.0.3     2022-04-02 [1] CRAN (R 4.2.0)
## Rsamtools         2.12.0    2022-04-26 [1] Bioconductor
## rstudioapi        0.14      2022-08-22 [1] CRAN (R 4.2.1)
## rtracklayer       1.57.0    2022-06-16 [1] Github (lawremi/rtracklayer@2bb0b40)
## rvest             1.0.3     2022-08-19 [1] CRAN (R 4.2.1)
## S4Vectors         0.34.0    2022-04-26 [1] Bioconductor
## scales            1.2.1     2022-08-20 [1] CRAN (R 4.2.1)
## sessioninfo       1.2.2     2021-12-06 [1] CRAN (R 4.2.0)
## statnet.common    4.7.0     2022-09-08 [1] CRAN (R 4.2.1)
## stringi           1.7.8     2022-07-11 [1] CRAN (R 4.2.1)
```

```
##  stringr              * 1.4.1     2022-08-20 [1] CRAN (R 4.2.1)
##  SummarizedExperiment   1.26.1    2022-04-29 [1] Bioconductor
##  syntenet             * 1.1.2     2022-11-17 [1] Bioconductor
##  tibble               * 3.1.8     2022-07-22 [1] CRAN (R 4.2.1)
##  tidyr                * 1.2.1     2022-09-08 [1] CRAN (R 4.2.1)
##  tidyselect             1.2.0     2022-10-10 [1] CRAN (R 4.2.1)
##  tidytree               0.4.1     2022-09-26 [1] CRAN (R 4.2.1)
##  tidyverse            * 1.3.2     2022-07-18 [1] CRAN (R 4.2.1)
##  treeio                 1.23.0    2022-11-10 [1] Github (GuangchuangYu/treeio@db85803)
##  tzdb                   0.3.0     2022-03-28 [1] CRAN (R 4.2.0)
##  utf8                   1.2.2     2021-07-24 [1] CRAN (R 4.2.0)
##  vctrs                  0.5.0     2022-10-22 [1] CRAN (R 4.2.1)
##  vroom                  1.6.0     2022-09-30 [1] CRAN (R 4.2.1)
##  withr                  2.5.0     2022-03-03 [1] CRAN (R 4.2.0)
##  xfun                   0.33      2022-09-12 [1] CRAN (R 4.2.1)
##  XML                    3.99-0.11 2022-10-03 [1] CRAN (R 4.2.1)
##  xml2                   1.3.3     2021-11-30 [1] CRAN (R 4.2.0)
##  XVector                0.36.0    2022-04-26 [1] Bioconductor
##  yaml                   2.3.5     2022-02-21 [1] CRAN (R 4.2.0)
##  yulab.utils            0.0.5     2022-06-30 [1] CRAN (R 4.2.1)
##  zlibbioc               1.42.0    2022-04-26 [1] Bioconductor
##
##  [1] /home/faalm/R/x86_64-pc-linux-gnu-library/4.2
##  [2] /usr/local/lib/R/site-library
##  [3] /usr/lib/R/site-library
##  [4] /usr/lib/R/library
##
## ------------------------------------------------------------------------------
```

References

Van Bel, Michiel, Tim Diels, Emmelien Vancaester, Lukasz Kreft, Alexander Botzki, Yves Van de Peer, Frederik Coppens, and Klaas Vandepoele. 2018. "PLAZA 4.0: An Integrative Resource for Functional, Evolutionary and Comparative Plant Genomics." *Nucleic Acids Research* 46 (D1): D1190–96.

Zhao, Tao, and M Eric Schranz. 2019. "Network-Based Microsynteny Analysis Identifies Major Differences and Genomic Outliers in Mammalian and Angiosperm Genomes." *Proceedings of the National Academy of Sciences* 116 (6): 2165–74.

Zhao, Tao, Arthur Zwaenepoel, Jia-Yu Xue, Shu-Min Kao, Zhen Li, M Eric Schranz, and Yves Van de Peer. 2021. "Whole-Genome Microsynteny-Based Phylogeny of Angiosperms." *Nature Communications* 12 (1): 1–14.
